# Attractiveness Is Multimodal: Beauty Is Also in the Nose and Ear of the Beholder

**DOI:** 10.3389/fpsyg.2017.00778

**Published:** 2017-05-18

**Authors:** Agata Groyecka, Katarzyna Pisanski, Agnieszka Sorokowska, Jan Havlíček, Maciej Karwowski, David Puts, S. Craig Roberts, Piotr Sorokowski

**Affiliations:** ^1^Institute of Psychology, University of WroclawWroclaw, Poland; ^2^Mammal Vocal Communication and Cognition Research Group, School of Psychology, University of SussexSussex, United Kingdom; ^3^Department of Psychotherapy and Psychosomatic Medicine, Technische Universität DresdenDresden, Germany; ^4^Department of Zoology, Faculty of Science, Charles UniversityPrague, Czechia; ^5^The Maria Grzegorzewska UniversityWarsaw, Poland; ^6^Department of Anthropology–Center for Brain, Behavior, and Cognition–Center for Human Evolution and Diversity, The Pennsylvania State University, University ParkPA, United States; ^7^Division of Psychology, University of StirlingStirling, United Kingdom

**Keywords:** physical attractiveness, smell, voice, multimodal perception, olfactory cues, acoustic cues

## Abstract

Attractiveness plays a central role in human non-verbal communication and has been broadly examined in diverse subfields of contemporary psychology. Researchers have garnered compelling evidence in support of the evolutionary functions of physical attractiveness and its role in our daily lives, while at the same time, having largely ignored the significant contribution of non-visual modalities and the relationships among them. Acoustic and olfactory cues can, separately or in combination, strongly influence the perceived attractiveness of an individual and therefore attitudes and actions toward that person. Here, we discuss the relative importance of visual, auditory and olfactory traits in judgments of attractiveness, and review neural and behavioral studies that support the highly complex and multimodal nature of person perception. Further, we discuss three alternative evolutionary hypotheses aimed at explaining the function of multiple indices of attractiveness. In this review, we provide several lines of evidence supporting the importance of the voice, body odor, and facial and body appearance in the perception of attractiveness and mate preferences, and therefore the critical need to incorporate cross-modal perception and multisensory integration into future research on human physical attractiveness.

## Introduction

Physical attractiveness plays a central role in the assessment of human mate value. This has made it a widely examined topic in contemporary psychology and biology. The variables that determine mate value, such as health, sexual maturity and reproductive potential, are often not directly observable. However, numerous studies have shown that these indices of mate value predict measures and ratings of physical attractiveness (for review see, Grammer et al., 2003). Sexual selection is therefore thought to have shaped psychological mechanisms whose function it is to extract and process information related to good health and reproductive ability (Singh and Randall, 2007). While physical attractiveness has been extensively examined in a mating context, attractiveness also plays an important role in various non-sexual social contexts such as friendship formation (Wang et al., 2010), school settings (Lerner and Lerner, 1977), and job interviews (Watkins and Johnston, 2000). It has also been examined as a potential risk factor for various mental disorders (Davis et al., 2000).

## Attractiveness is Multimodal

Researchers have garnered compelling evidence in support of the evolutionary functions of physical attractiveness and its role in our daily lives, although the overwhelming focus has been on the contribution of visual cues (Eagly et al., 1991; Langlois et al., 2000; Weeden and Sabini, 2005; Maestripieri et al., 2016; but see Puts et al., 2012). However, while visual cues are indeed strong predictors of overall attractiveness judgments (Douglas and Shepard, 1998; Sorokowski et al., 2013), attractiveness is also influenced by a person’s voice (for reviews see, Hill and Puts, 2016; Pisanski and Feinberg, 2017) and scent (Roberts et al., 2011). Together, vision, audition, and olfaction form the key telereceptive senses that process both proximal and distant sensory information in the external environment, and which, in combination, increase the efficiency of our actions and reactions when processing critical social cues (Aglioti and Pazzaglia, 2011). In contrast to other senses (taste and touch), people can form first impressions of others based on their visual appearance, voice or smell even at some distance, without engaging the person’s will or awareness. In this paper, we argue that a more balanced approach that integrates research across these three modalities will provide stronger evidence regarding the complex factors underlying human attractiveness and the degree to which attractiveness influences human life.

Several empirical studies demonstrate that the perception of attractiveness is multimodal. At the neural level, multiple modalities in person perception are integrated in the superior temporal sulcus (STS; Campanella and Belin, 2007). At a functional level, facial, vocal, and olfactory attractiveness have all been linked to traits indicative of sex hormone levels and health (e.g., Rantala et al., 2006; Feinberg, 2008; Puts et al., 2012). Indeed, attractiveness judgments often co-vary across modalities (Rikowski and Grammer, 1999; Saxton et al., 2009; Hughes and Miller, 2016), although these cross-modal relationships can differ by sex (Currie and Little, 2009; O’Connor et al., 2013; Hill et al., 2016; Valentova et al., 2017). Despite a growing body of research concerning the attractiveness of vocal and olfactory cues, these studies remain scarce compared to the vast number of studies examining visually assessed physical attractiveness, most of which focus on the face.

The amount of information one can gauge about a person solely from her or his scent and voice is impressive. For example, humans can use olfactory cues present in body odor to assess sex (Schleidt et al., 1981), personality (Sorokowska, 2013a) including dominance (Havlíček et al., 2005), actual fertility (Gildersleeve et al., 2012), diet (Fialová et al., 2013), genetic compatibility (Havlíček and Roberts, 2009), health status (Moshkin et al., 2012), and age (Mitro et al., 2012). Humans also have the capacity to recognize kin via body odor (Weisfeld et al., 2003; Ferdenzi et al., 2010), which may be important in mate choice in order to avoid inbreeding. Similarly, vocal cues allow others to make relatively accurate judgments about, for example, another person’s sex (Lass et al., 1976), age (Bruckert et al., 2006), dominance (Puts et al., 2007), cooperativeness (e.g., Knowles and Little, 2016), emotional state (Scherer, 1986), physical strength (Sell et al., 2010), body size (Pisanski et al., 2014a) and actual fertility (Pipitone and Gallup, 2008; Puts et al., 2013).

Given that ecologically relevant information is perceptually available in others’ voices and body odors, it is likely that voice and odor play a salient role in our everyday decision-making, and that utilizing and integrating information from the visual, acoustic and olfactory channels may improve social communication. Thus, the importance of modalities other than vision in social perception should not be neglected in scientific research.

While multisensory integration in human perception is uncontroversial, the number of researchers examining this phenomenon in social communication remains relatively small, and the mechanisms underpinning it remain unclear. Brain imaging studies suggest that the neural response to combined visual–olfactory cues in the right middle temporal cortex and left superior parietal cortex is super-additive – higher than the sum of visual and olfactory cues presented in isolation (Royet et al., 2013). There is also growing evidence that the STS region of the brain preferentially processes social information garnered from both the face (for review see, Allison et al., 2000) and the voice (Belin et al., 2000). This suggests that the human brain may be ‘hard wired’ to process faces and voices differently from other visual and auditory stimuli. Perceptual experiments examining visual and auditory adaptation effects further suggest that mental representations of faces and voices overlap cross-modally (Little et al., 2013).

## Evolutionary Significance of Multimodal Indices of Attractiveness

There are several evolutionary explanations regarding the potential adaptive functions of multisensory integration in person perception. Extrapolating from work on multiple signaling in animals (Möller and Pomiankowski, 1993), the multiple message hypothesis proposes that each signal or cue reflects a unique and independent property of an individual’s overall condition or quality. Alternatively, according to the redundant signal (or ‘back up’) hypothesis (Zuk et al., 1992; Thornhill and Grammer, 1999), each trait provides similar and overlapping information. Following this model, individuals pay attention to several traits or modalities because, in combination, multiple traits provide a better estimate of general condition than any single trait. The unreliable signal hypothesis (Möller and Pomiankowski, 1993) argues that some traits are unreliable indicators of overall condition and are only maintained because they are relatively uncostly to produce and because there is a weak preference for them. Finally, fitness indicator theory (Miller, 1998, 2000a) posits that an individual’s genetic quality is expressed by combining various phenotypic traits that each indicate fertility and health. The theory states that these signals can be perceived at a distance through different channels (vision, audition, or olfaction) and are useful not only for attracting mates, but also for deterring predators and intimidating rivals (Miller, 2000a). The considerations about indicators of fitness go far beyond physical traits and include, for example, intelligence and humor (e.g., Miller, 2000b; Prokosch et al., 2004; Howrigan and MacDonald, 2008; Sefcek and Figueredo, 2010). These theories are not all mutually exclusive and the degree to which they apply to multiple indicators of attractiveness is likely to vary across traits.

Some studies suggest that information gauged from multiple modalities can have both independent and additive effects on judgments of attractiveness, such that voices, faces, bodies, and body odors can provide some partly redundant information about mate quality, but also some non-redundant information. For instance, faces and bodies appear to contribute independently to overall attributions of attractiveness, with faces explaining significantly more of the variation for both men and women than bodies (Peters et al., 2007) suggesting multiple signaling, however, only in terms of one modality. In contrast, combining an attractive face with an attractive voice or scent can result in higher overall judgments of attractiveness than presenting any modality alone (Ferdenzi et al., 2016), which can be interpreted as support for the redundant signal hypotheses. Yet, even in the absence of visual cues, an attractive voice (Pisanski and Feinberg, 2017) or an attractive body odor (Gueguen, 2001; Sorokowska, 2013b) can elicit prosocial behavior or generate positive impressions in others, and can independently predict individual differences in reproductive and socioeconomic success (e.g., Puts et al., 2012). Thus, non-visual indices of attractiveness may account for additional variation in the ‘attractiveness premium’ that is unaccounted for by measuring visual attractiveness alone (see also Saxton et al., 2009).

## The Complex Nature of Multimodal Perception

Studies examining interactions among the modalities underscore the inevitable complexity of multimodal sensory integration when it comes to judging attractiveness. For example, men’s preferences for relative femininity in women’s faces and voices correlate, yet this cross-modal interaction does not always generalize to men’s assessments of other men (O’Connor et al., 2013). Women, on the other hand, prefer an intermediate level of overall masculinity and appear to achieve an optimal average level of this dimension either by preferring an intermediate level of masculinity for each modality or trait (e.g., body appearance or voice acoustics), or employing flexible cross-modal trade-offs (e.g., they might prefer less masculine bodies in men with more masculine voices, and vice versa) (Hill et al., 2013). Further, in studies of genetic complementarity between partners, an intermediate level of genetic dissimilarity is usually optimal. While people tend to find faces of others with genotypes similar to their own most attractive, they prefer the odors of those with dissimilar genotypes. As such, face and odor preferences might be used in tandem to filter out unsuitable partners at either extreme to achieve optimal complementarity (Roberts et al., 2005; but see Winternitz et al., 2017).

The relative importance of each modality might also shift dynamically during relationship formation. For example, visual and vocal characteristics are likely to be more important early on, whereas odor requires closer and more intimate physical contact. Potential mates may utilize physical appearance as a first-pass screen, while smell potentially imparts additional information during subsequent inspection. Other shifts in preferences may occur across women’s menstrual cycles. It has been shown that women’s preferences for men’s voices (Pisanski et al., 2014b), odors (Havlíček et al., 2005) and faces (Penton-Voak et al., 1999) peak around the time of ovulation, however, it remains unknown whether such cyclic effects generalize to cross-modal integration (for review see, Havlíček et al., 2015).

The relative importance of various traits or modalities may also vary contextually. For instance, Currie and Little (2009) as well as Confer et al. (2010) showed that women’s bodies are relatively more important to men’s judgments of attractiveness in a short-term relationship context, whereas facial appearance becomes more critical in a long-term relationship context. This difference is less apparent in women’s judgments of men’s traits. Men and women also differ in the relative importance they ascribe to various attributes of a potential mate. For example, while men rely more on visual attributes, women pay more attention to olfactory cues (Havlíček et al., 2008).

## Conclusion

The complexity of what people perceive as attractive highlights the need for more research on the multimodal nature of person perception, as challenging as this may be. In addition to studying each modality as if it exists independently of the others (which in the real world it most often does not), researchers have focused disproportionately on visual indicators of attractiveness, underplaying the influence of scent and voice. In a recent and relatively broad theoretical review of prosocial biases in favor of attractive people, Maestripieri et al. (2016) refer directly to the attractiveness of voices and scents only once (see also Gangestad and Scheyd, 2005). This is true of older reviews as well (e.g., Gangestad and Scheyd, 2005), and some reviews do not mention these modalities at all (e.g., Buggio et al., 2012). Similarly, studies examining the correlates of physical attractiveness (e.g., social competence, professional success), focus mainly on its visual aspects. We cannot ignore the important contribution that cross-modal research can offer to our understanding of the evolutionary origin and social functions of attractiveness and mate preferences. We hope that more researchers interested in attractiveness (and in person perception more broadly) will integrate visual and non-visual markers of attractiveness in their research, and in doing so contribute to a better understanding of multisensory integration and the role that beauty plays in our everyday lives.

## Author Contributions

Idea of the review: PS, KP, AS, and MK. Wrote the manuscript: AG, KP, AS, JH, MK, DP, SR, and PS.

## Conflict of Interest Statement

The authors declare that the research was conducted in the absence of any commercial or financial relationships that could be construed as a potential conflict of interest.
